# The NOP-1 peptide derived from the central regulator of ethylene signaling EIN2 delays floral senescence in cut flowers

**DOI:** 10.1038/s41598-018-37571-x

**Published:** 2019-02-04

**Authors:** Claudia Hoppen, Lena Müller, Anna Christina Albrecht, Georg Groth

**Affiliations:** Institute of Biochemical Plant Physiology and Bioeconomy Science Center (BioSC), Heinrich Heine University Düsseldorf, Düsseldorf, Germany

## Abstract

The plant hormone ethylene was identified as important triggering factor and primary regulator of flower senescence in many species. Consequently, application of chemical inhibitors of ethylene biosynthesis and action is used to extend the longevity of ethylene-sensitive flowers. Here, we show that the peptide NOP-1, a biological derived from the nuclear localization signal of ethylene regulator EIN2 tightly binds to the ethylene receptor of carnation plants - a model to study flower senescence. When applied on cut flowers the peptide biological delays petal senescence similar to previously identified and currently used chemical inhibitors, but offers significant advances to these chemicals in biodegradability, sustainability and ecotoxicity. Our bioinformatic analysis of a wide range of ethylene receptors indicates complete sequence conservation of the anticipated NOP-1 binding site in flower species supporting a widespread use of the peptide on flowering ornamentals to delay senescence and decay in cut flowers. We anticipate our innovative approach to extend flower longevity by a new class of biomolecules such as peptides, peptide analogues and peptide mimetics will significantly advance our technological capability to delay flower senescence and expand vase-life of cut flowers in a sustainable and environmentally friendly manner.

## Introduction

Flower senescence is a tightly regulated developmental process that plays a crucial role in the overall reproductive strategy of many plants. Initial work on this developmental process was motivated primarily by the commercial interest in increasing the life time of cut flowers which according to import statistics reported to United Nations (UN) show an overall world trade value of around 4 billion USD. Europe (66.7%), the US (19.3%) and Japan (10.7%) form the three most important floriculture consumption regions in this market. Globally, both, traders and customers, demand for cut flowers with a long vase-life no matter whether those have already experienced long-distance transport from their cultivation areas in Latin America or Eastern Africa. Accordingly, chemicals or processes that decelerate or delay flower senescence are of broad social and economic interest.

Identification of the plant hormone ethylene as primary regulator of carnation senescence^1^ and the drastic extension of life of petals after treatment of cut flowers by inhibitors of ethylene biosynthesis^2^ or ethylene action inhibitors resulted in a range of commercial treatments to extend the vase life of cut flowers^3^. Small molecule inhibitors amino-ethoxyvinyl glycine (AVG) and amino-oxyacetic acid (AOA) which interfere with ethylene biosynthesis have been shown effective in blocking ethylene production that accompanies senescence. Consequently, a number of commercial products based on these chemicals have been introduced to the market to delay floral senescence and abscission^4–8^. However, these inhibitors are ineffective in preventing the effects of exogenous ethylene on flower senescence during transport and storage. Hence, more commercial interest was received for inhibitors blocking ethylene perception such as silver ions^3^ and the cyclopropene derivative 1-MCP^9^. Silver salts which were shown to decrease the number of active ethylene binding sites and alter signal output of the receptors^10^ have been an industry standard for preventing ethylene action in ornamentals for decades. But nowadays the use of the heavy metal pollutant is banned in many countries due to serious concerns about its potential as a groundwater pollutant. In recent years, the cyclic olefine 1-MCP has been widely adopted in the ornamental plant industry as a non-toxic alternative to silver salts, although it does not control senescence for as long as silver ions when applied in a single treatment. However, repetitive treatments with the gaseous inhibitor resulted in a marked increase in vase life and efficiently blocked floral senescence of cut flower^11^. Hence, 1-MCP today is widely used as ethylene action inhibitor at a wide range of ornamental plants^12^ which due to the gaseous character of the inhibitor are treated in enclosed, gas-tight areas. The inhibitor was shown to be highly efficient at very small concentrations^12^, although treatment depends on temperature achieving complete inhibition at 20 °C, but almost no inhibition at 0 °C^13^. In addition to chemical treatments, transgenic approaches targeting ethylene biosynthesis or ethylene signaling were applied to extend flower vase life^14,15^. Overall, each method comes with its own chances and drawbacks^16^. Hence, a sustainable and easy-to-use method to improve longevity of cut flower has not been found yet.

The different strategies developed in the past to delay floral senescence have been used on a wide range of ornamental plants. However, among ethylene-sensitive flowers carnations are probably the most studied model system^13,17,18^ as they are highly sensitive and rapidly respond to the plant hormone by clear physiological and morphological changes which can be studied even on individual petals^19^.

Much of the current knowledge on ethylene perception and transduction has been established by physiological, biochemical and genetic studies in the small crucifer weed *Arabidopsis*, and crops such as tomato or rice. These studies clarified that ethylene is perceived by a family of receptor proteins (ETRs), which form homo- and heterodimers at the ER membrane^20–23^. Although the exact output of the receptors is still obscure, genetic and biochemical studies established that in the absence of ethylene, a receptor associated kinase (CTR1) phosphorylates a central regulator of the signaling cascade (EIN2). Conversely, in the presence of ethylene the CTR1 kinase is inactivated leading to dephosphorylation of EIN2^24^. In turn, the EIN2 C-terminal domain (aa 462–1294) containing a highly conserved nuclear localization signal^25,26^, is processed by a so far unknown protease and translocated to the nucleus^24,26,27^ where it activates a variety of ethylene response genes and phenotypes. Recent studies in our lab propose an efficient way to interfere with ethylene signaling based on a yet unknown function of the nuclear localization signal (NLS) in EIN2. Peptides mimicking this NLS motif were shown to affect ethylene responses in *Arabidopsis* and also function as potent inhibitor of tomato fruit ripening^28,29^.

## Results and Discussion

**Figure 1:**
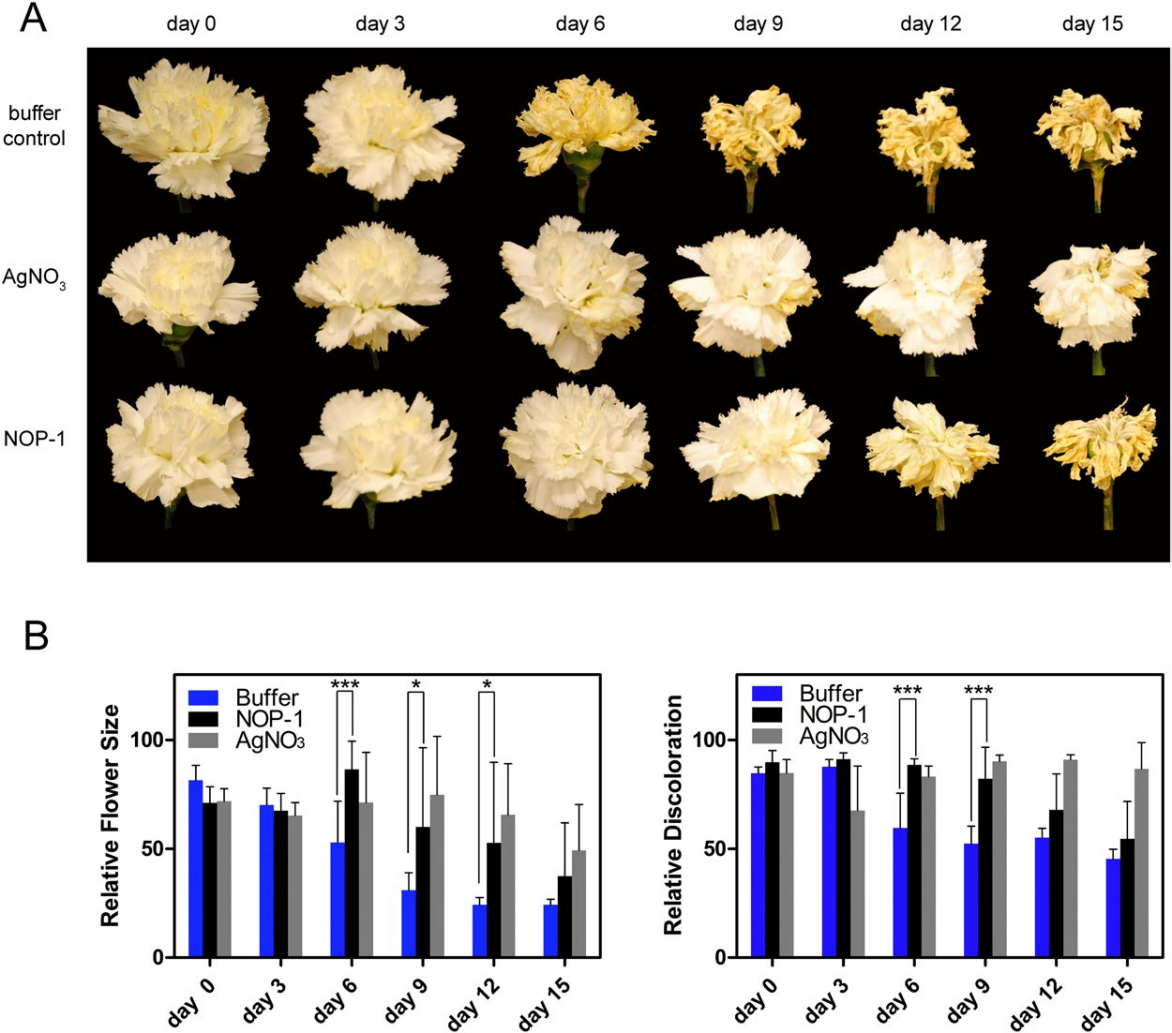
Effect of small molecule inhibitors of ethylene signaling on flower senescence of cut carnations. (**A**) Visual images of carnation cut flowers treated with senescence inhibitor silver nitrate or peptide NOP-1. Representative photos of carnation petals treated with 1 mM silver nitrate or 0.5 mM NOP-1 on DAT 0, 3, 6, 9, 12 and 15. Controls treated with buffer only are depicted in the upper row. (**B**) Quantitative analysis of flower size (left) and petal color change (right) of carnation flowers after treatment with silver salt, NOP-1 peptide or buffer-only. Asterisks indicate significance level (***99%, *90%) using Student’s t-test.

**Figure 2:**
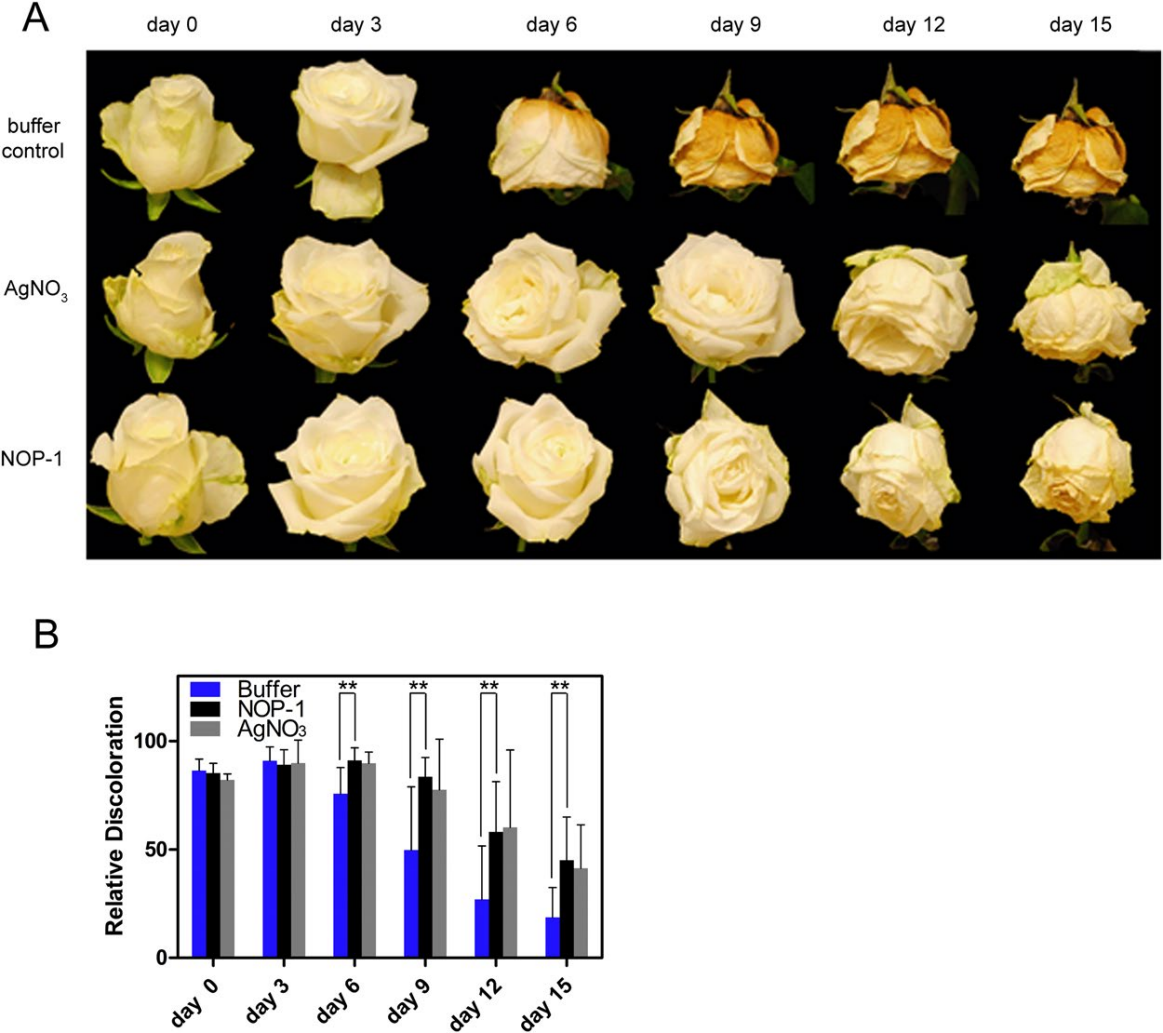
Effect of small molecule inhibitors of ethylene signaling on flower senescence of cut roses. (**A**) Images of cut roses treated with senescence inhibitors silver nitrate or NOP-1, respectively. Representative photos of rose ornamentals treated with 1 mM silver nitrate or 0.5 mM NOP-1 on DAT 0, 3, 6, 9, 12 and 15. Controls treated with buffer only are depicted in the upper row. (**B**) Quantitative analysis of color change in petals after treatment with silver salt, NOP-1 peptide or buffer-only. Asterisks indicate significance level (**95%) using Student’s t-test.

To clarify whether NOP-1 also has the potential to serve as an inhibitor of senescence in cut flowers, we applied the peptide on cut carnations (*Dianthus caryophyllus*) and analyzed the effect on floral senescence in this model ethylene response system^9,13,17,30^. In analogy to treatment with the chemical senescence inhibitor STS containing silver ions as active ingredient which target plant ethylene receptors^31^ the peptide was directly supplied to the vase water. Senescence was monitored visually throughout the experiment and quantified by changes observed in petal size and/or color (Fig. 1A,B). While controls with no additions to vase water show a clear senescence phenotype of petal inrolling and petal fading after 6 days (Fig. 1A, upper panel), the addition of 1 mM silver salt delays the appearance of these morphological manifestations by several days and petals show almost no signs of senescence after 15 days (Fig. 1A, middle panel). Similarly, addition of NOP-1 peptide at 0.5 mM concentration clearly delays the onset of petal senescence and significantly increases flower longevity, although the delay is shorter than observed with the potent silver salt inhibitor (Fig. 1A, lower panel). To further substantiate the effect of the peptide biological on floral senescence observed with the carnation model system, we used roses as another species of highly ethylene sensitive ornamental plants. Similar to carnations untreated roses showed obvious signs of senescence such as wilting and petal bending by day 6 (Fig. 2A, upper panel). The untreated controls completely ended their vase life on day 9 due to these morphological changes. In contrast, cut roses treated with the silver salt inhibitor (Fig. 2A, middle panel) and flowers treated with the NOP-1 peptide (Fig. 2A, lower panel) showed a prolonged vase life of about 6–8 days relative to buffer controls. While the silver salt chemical inhibitor was noticeably more efficient in extending the vase life of cut carnations than the peptide biological (Fig. 1B), the difference on flower longevity between both treatments is less pronounced for cut roses (Fig. 2B). Nevertheless, on the entire trial period, NOP-1 was less effective than the silver treatment similar to what is seen for carnation. Noteworthy, no beneficial effect on flower longevity was observed by multiple treatments with the NOP-1 peptide in contrast to studies with the gaseous ethylene antagonist 1-MCP which significantly delayed flower senescence when applied on a daily basis^11^. Hence, further testing remains to be done to uncover the full potential of the peptide biological in order to identify optimum treatment conditions (concentration, duration, temperature) and strategies for efficient application.

**Figure 3:**
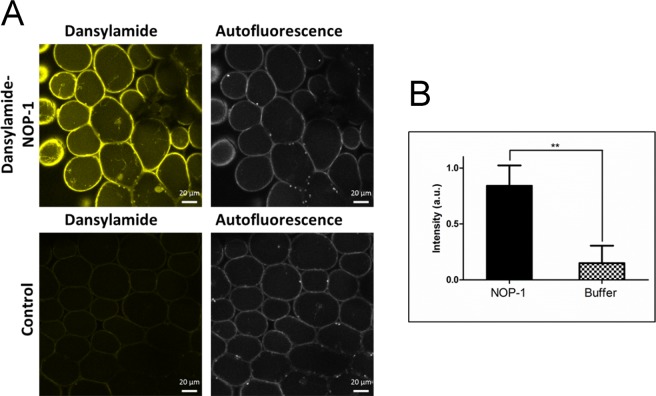
Detection of uptake and transport of fluorescently-labelled NOP-1 to petals. (**A**) Confocal imaging of carnation petals from plants watered for 3 days with a solution of 1 mM dansylamide-labeled NOP-1 or buffer-only, respectively. Uptake of the NOP-1 peptide from the vase water to the petal cells was visualized by the dansyl fluorophore. (**B**) Dansyl content in treated and untreated petals quantified by UV absorption spectroscopy (right).

To demonstrate uptake of the peptide from the vase water and vascular transport to petals fluorescently labeled NOP-1 was used. To this end, dansylated NOP-1 was added directly to the vase water of cut carnation flowers at 1 mM concentration. Confocal microscopy and spectroscopic analysis of mock and NOP-1 treated flowers clearly demonstrate significant accumulation of the peptide in petal cells (Fig. 3). Consequently, this also verifies efficient transport of the NOP-1 peptide across the vascular system. No fluorescence was detected in mock controls. Moreover, we noted that there is no indication of cytotoxic effects on carnation cells by NOP-1 or dansylated NOP-1.

**Figure 4:**
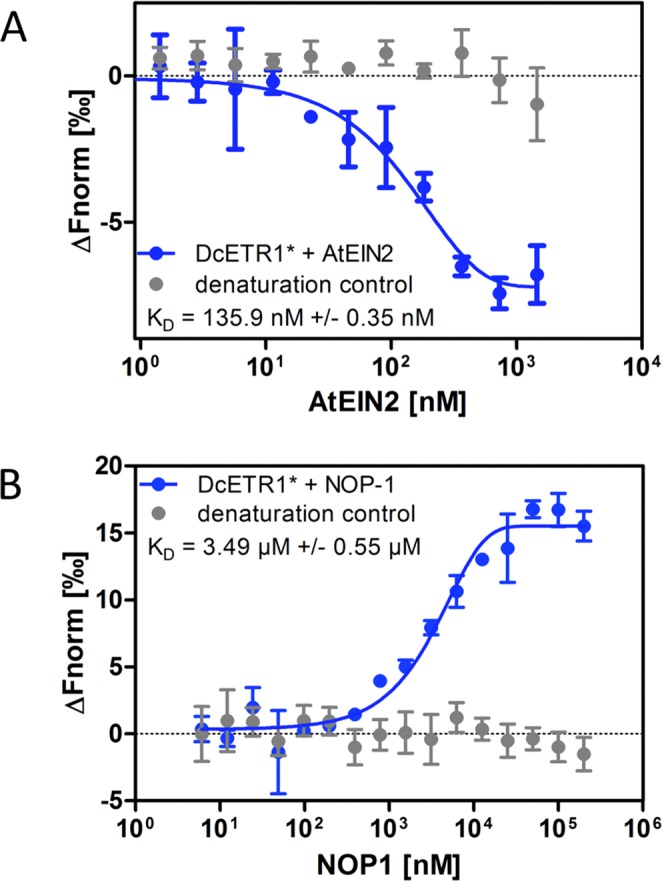
Molecular binding studies of carnation ethylene receptor DcETR1. (**A**) Microscale Thermophoresis (MST) binding studies using recombinant carnation ethylene receptor DcETR1 and AtEIN2. Titration of unlabeled AtEIN2 to labeled DcETR1 (•) revealed binding of both proteins at a dissociation constant (K_D_) of 135 nM ± 0.35 nM, indicating a tight interaction between DcETR1 and AtEIN2. (**B**) MST binding studies on DcETR1 and NOP-1. Titration of unlabeled NOP-1 to labeled DcETR1 (•) resulted in a K_D_ of 3.49 µM ± 0.55 µM. Asterisks (*) indicate the labeling of DcETR1 with AlexaFluor488-NHS. Negative controls using chemically denatured DcETR1 shows no interaction with the EIN2 protein or the NOP-1 peptide (•).

**Figure 5:**
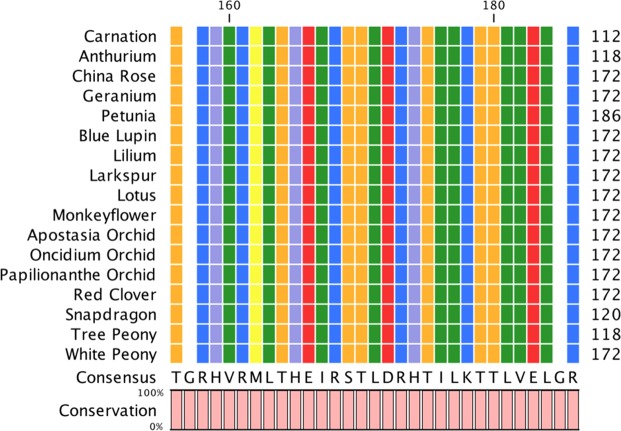
Conservation of NOP-1 binding site in ethylene receptors. Alignment of the proposed NOP-1 binding site in the GAF domain of ETR1 receptors from flowering ornamentals used as cut flowers. For accession numbers and the complete alignment see the Material and Methods section and Supplemental Data Fig. S3.

To elucidate the mode of action of NOP-1 on flower senescence and to determine whether the peptide biological works by the same molecular mechanism as determined for NOP-1 controlled ripening delay, we initiated interaction studies of the carnation ethylene receptor DcETR1, the central regulator of the ethylene signaling cascade EIN2 and the NOP-1 peptide. To this end, DcETR1 was cloned in *E*. *coli*, expressed and purified according to protocols established in our lab^32^ for ethylene receptors from *Arabidopsis thaliana*, *Physcomitrella patens*, tomato and apple (Supplementary Fig. S1). Functional folding of the recombinant purified proteins was demonstrated by CD spectroscopy (Supplementary Fig. S2) and purified DcETR1 was labeled with fluorescent dye AlexaFluor488. To determine binding affinity and dissociation constants of the purified recombinant proteins and the NOP-1 peptide we used microscale thermophoresis. In these experiments we observed tight binding of the DcETR1 receptor to the EIN2 ethylene central regulator (Fig. 4A, K_D_ = 135.9 nM ± 0.35 nM) as previously observed for the interaction of the corresponding proteins from *Arabidopsis* and tomato^29,33^. These data indicate a high affinity and central protein protein interaction (PPI) common in ethylene signaling among plant species. Consequently, targeting this interaction by small-molecule modulators has the potential to shut down ethylene signaling and related plant responses. Therefore, we tested binding of the ripening delaying peptide NOP-1 identified in previous studies to target the ETR1-EIN2 complex with our purified carnation receptor DcETR1. Our data on the carnation protein confirm binding of the peptide biological at similar affinities (Fig. 4B, K_D_ = 3.49 µM ± 0.55 µM) as earlier observed for *Arabidopsis* and tomato receptors^29,34^. The precise interaction site of NOP-1 at the *Arabidopsis* ETR1 receptor was recently resolved in our lab by molecular and computational studies^34^. In these studies, we uncovered binding of the peptide biological at the GAF domain of the receptor. Based on the resolved binding site and binding mode we proposed that the bound peptide arrests intra- and intermolecular downstream signaling in the receptor resulting in the observed ripening inhibition. To verify that the NOP-1 binding site identified in the *Arabidopspis* ETR1 receptor is also present in ethylene receptors of cut flowers and ornamental plants, we compared the corresponding sequence (aa 143–174 in *Arabidopsis*) in 17 flowering ornamentals widely used as cut flowers. Remarkably, all tested varieties show an identical protein sequence (Fig. 5). The extreme conservation found in this part of the receptor (see also Supplementary Fig. S3) further emphasizes a potential critical role of this element in downstream signal transfer. Moreover, the exact match of this patch in the receptors of different flower species supports a general usage of NOP-1 as a senescence inhibitor in a variety of cut flowers without the need to genetically engineer ornamental plants in ethylene biosynthesis or perception.

Compared to current approaches to expand the vase-life of cut flowers, NOP-1 offers a number of advantages, such as application as aqueous solution, ultimate biodegradability, sustainability and low ecotoxicity due to the lack of adverse effects to the environment. In contrast, silver salts although they offer long lasting senescence protection have various drawbacks. As a heavy metal they are highly toxic and harmful to the environment, persist in soil and groundwater for long periods and pollute drinking water^35^. 1-MCP, another senescence inhibitor used to extend longevity of cut flowers, lacks most of these disadvantages, is easy to dispose and safe, but as a gas is active by fumigation only complicating its usage on cut flowers. Hence, the peptide biological NOP-1 combines the strengths of current senescence inhibitors and thereby offers an alternative and sustainable approach to prolong the vase-life of cut flowers. To further improve the effect on the vase-life of cut flowers, additional studies on more flower species and variants of the NOP-1 biological (peptide length, stability, modifications) are needed. Nevertheless, our present work highlights a novel way to address flower longevity and presents future opportunities to control senescence delay in cut flowers.

## Material and Methods

### Treatment of cut flowers with NOP-1 peptide

Cut flowers of carnations (*Dianthus caryophyllus*) and rose (*Rosa hybrida*), graded for marketable quality, were obtained from a local flower producer. Upon arrival in the laboratory, the cut flower stems were re-cut to a length of approximately 23 cm. Flowers were treated with NOP-1 by adding the peptide dissolved in 50 mM Tris/Acetic acid pH 8 at a final concentration of 0.5 mM directly to the vase reservoir. Cut carnations placed in 50 mM Tris/Acetic acid pH 8 were used as mock control. Flowers with 1 mM of senescence inhibitor AgNO_3_ added to the buffer served as positive control. Six flowers were used for each treatment to evaluate vase life throughout the experiment. Progression of senescence was recorded photographically for a period of 15 days. Images were acquired using fixed camera setting and set-up to ensure high data quality and reliability. Changes in petal size and color were quantified using ImageJ (32 bit v1.51t.). Relative discoloration of petals was quantified by drawing multiple lines across each flower (petals only) and calculating the average values for red (R), green (G) and blue (B). Data shown represent the values for blue as these showed the most significant difference between fresh and senescent flowers. Relative size of flowers was quantified using a macro that specifically recognizes the flower (petals + sepals) but not the flower stem with flower size in pixel² as the output. All Data are normalized to the maximum.

### Uptake experiments with fluorescently labelled NOP-1

Carnations were watered with 1 mM Dansylamide-labeled NOP-1 or tap water only, respectively. Dansylamide fluorescence was imaged after 3 days using a Zeiss LSM780 confocal microscope (Dansylamide: λ_ex_ 405 nm, λ_em_ 500–550 nm; auto fluorescence: λ_ex_ 488 nm, λ_em_ 510–650 nm). To estimate NOP-1 uptake 500 mg petals of treated and untreated carnations (triplicates) were homogenized in 500 µl water 3 days after treatment. Samples were centrifuged at 14.100 × g for 30 min. The resulting clear cell lysate was diluted 1:100 and absorbance (250 nm – 400 nm) was recorded using a DU800 spectrophotometer (Beckman Coulter, Krefeld). Absorbance at 330 nm (λ_ex_ (max) of dansylamide) was divided by the absorbance at 280 nm (proteins). Based on these data the difference between treated and untreated carnation flowers was calculated. Data shown are normalized to the maximum.

### Cloning of *Dianthus caryophyllus* ethylene receptor ETR1 into expression vector pET16b

According to the published sequence (Carnation DB: Dca62022.1), full length codon optimized cDNA sequence encoding *Dianthus caryophyllus* ethylene receptor DcETR1 was ordered at GenScript United States. Vector construction of pET16b vector (Novagen, Madison, WI, United States) was performed by Gibson Assembly. The final construct carries an ampicillin resistance gene, a deca-histidine tag and the target DNA sequence of DcETR1. For linearized vector amplification oligonucleotides 5′-GGATCCGGCTGCTAACAAAGC-3′ as forward primer and 5′-ATGACGACCTTCGATATGGC-3′ as reverse primer were used. The target gene was amplified using 5′-ATCGAAGGTCGTCATATGGAGAGCTGCAACTGCATC-3′ as forward primer and 5′-TTAGCAGCCGGATCCTTACTTCGGCATCGGCTCAAA-3′ as reverse primer. Fragments were fused to the final circular expression vector by adding Gibson Assembly Master Mix including an exonuclease, a DNA polymerase and a ligase. The sample was incubated at 50 °C for 10 min and at 40 °C for 60 min. The assembled plasmid was transformed into *E*. *coli* XL 1-blue cells and sequenced at Seqlab (Göttingen, Germany).

### Expression of recombinant carnation ethylene receptor protein DcETR1 in *E*. *coli* strain C43 (DE3)

For the expression of recombinant DcETR1 2YT medium [1.6% (w/v) peptone, 1% (w/v) yeast extract and 0.5% (w/v) NaCl] added with 2% (v/v) ethanol and 100 µg/mL ampicillin was used. Expression plasmid pET16b_DcETR1 was transformed in *E*. *coli* strain C43 (DE3). Cells were grown at 30 °C to an OD_600_ = 0.4. Temperature was reduced to 16 °C and expression of DcETR1 was induced by adding 0.5 mM IPTG at OD_600_ = 0.6. The expression was stopped after 20 h and cells were harvested by centrifugation at 7000 × g and 4 °C for 15 min.

### Solubilization, purification and labeling of recombinant DcETR1 with AlexaFluor488-NHS

C43-cells containing expressed carnation ethylene receptor DcETR1 were resuspended in PBS with 20% (w/v) glycerol, 1 mM (w/v) DTT and 0.002% (w/v) PMSF (buffer P). Resuspended cells were broken using a Constants Cell Disruption System (Constant Systems, Daventry, United Kingdom) at 2.4 kbar and 4 °C. The cell lysate was centrifuged at 14,000 × g and 4 °C for 30 min. Afterwards the supernatant was centrifuged again at 40,000 × g and 4 °C for 30 min. The resulting membrane pellet was resuspended in buffer P and centrifuged at 34,000 × g and 4 °C for 30 min. For solubilization the pellet was resuspended in 50 mM Tris/HCl pH 8, 200 mM NaCl, 1.2% (w/v) FosCholine-16, 0.002% (w/v) PMSF (buffer S) and stirred for 1 h at 700 rpm and RT. Solubilized ethylene receptor protein DcETR1 was separated from membrane fragments by ultracentrifugation at 229,600 × g and 4 °C for 30 min. The resulting supernatant was applied to metal ion affinity chromatography. To this end, the protein solution was loaded on a 5 mL HisTrap FF column operated on a ÄKTAprime plus (both GE Healthcare Life Sciences) at 4 °C previously equilibrated with 50 mM Tris/HCl pH 8, 200 mM NaCl and 0.002% (w/v) PMSF (buffer A). The protein bound to the column was washed with 50 column volumes 50 mM Tris/HCl pH 8, 200 mM NaCl, 50 mM KCl, 20 mM MgCl_2_, 10 mM ATP and 0.002% (w/v) PMSF (buffer ATP). Then the column was washed with buffer A containing 50 mM imidazole and the protein was eluted in buffer A containing 250 mM imidazole. Purified DcETR1 was concentrated and buffer exchanged to 100 mM potassium phosphate, 300 mM NaCl, 0.015% (w/v) FosCholine-16 and 0.002% (w/v) PMSF (buffer M). For labeling DcETR1 was incubated with 2.5 fold concentration of AlexaFluor488-NHS for 30 min at RT. Buffer was changed again to 50 mM Tris/HCl pH 8, 300 mM NaCl, 5% (w/v) glycerol, 0.015% (w/v) FosCholine-16 and 0.002% (w/v) PMSF (buffer L).

### DcETR1 binding studies by Microscale Thermophoresis

Recombinant proteins DcETR1 and AtEIN2^479–1294^ were expressed, purified and labeled as described in the Supplementary Information (Figure S1) and in^29^. For binding studies DcETR1 and NOP-1 were dissolved in 100 mM potassium phosphate buffer, 300 mM NaCl and 0.015% (w/v) FosCholine-16. DcETR1 labelled with Alexa488 was used at a concentration of 50 nM. NOP-1 was diluted in 100 mM potassium phosphate buffer, 300 mM NaCl and 0.015% (w/v) FosCholine-16 from 100 µM to 3 nM. AtEIN2^479–1294^ was diluted in 50 mM potassium phosphate (pH 7.8), 300 mM NaCl, 0.015% (w/v) FosCholine-16 from 1457.5 nM to 0.7 nM. Samples were loaded into premium glass capillaries and thermophoresis was measured using a Monolith NT.115 (NanoTemper Technologies GmbH, München, Germany) with 50% (NOP-1) or 60% (AtEIN2) MST power. As negative control binding partners were chemically denatured using 2% (w/v) SDS and 40 mM DTT in the buffer used in the assay. All measurements were run in triplicates.

### Amino acid alignment of ethylene receptor 1

Amino acid sequences of 17 different species where aligned using CLC Sequence Viewer 8 (QIAGEN Bioinformatics, Hilden, Germany). Accession numbers of the sequences used are A0A2P6RRN0, A0A1J7HIG6, A0A2K3PFW0, Q9XH58, B6CUX7, A0A1U8BHB1, J9XY08, Q8GV14, A0A022RCX4, Q8W2M7, Q8SBD2, A0A1D1XH0, Q206Y9, A0A2I0A057, F6KPP8 and X2KXU7.

## Supplementary information

Supplementary Information

## Data Availability

The data that support the findings of this study are available from the corresponding author upon request.
