# Supplementary material for: The NOP-1 peptide derived from the central regulator of ethylene signaling EIN2 delays floral senescence in cut flowers
Source: Sci Rep. 2019 Feb 4;9:1287. doi: 10.1038/s41598-018-37571-x (PMC6361973; doi:10.1038/s41598-018-37571-x)
Supplement: Supplementary file 1 — Supplementary Information [file 41598_2018_37571_MOESM1_ESM.docx]

**Supplementary Information**

**The NOP-1 peptide derived from the central regulator of ethylene signaling EIN2 delays floral senescence in cut flowers**

Claudia Hoppen^1,†^, Lena Müller^1,†^, Anna Christina Albrecht^1^, and Georg Groth^1,*^

^1^Institute of Biochemical Plant Physiology and Bioeconomy Science Center (BioSC), Heinrich Heine University Düsseldorf, Düsseldorf, Germany

^†^C.H. and L.M. contributed equally to this work.

*Correspondence should be addressed to G.G., e-mail: [georg.groth@hhu.de](mailto:georg.groth@hhu.de)

**Figure S1**

**
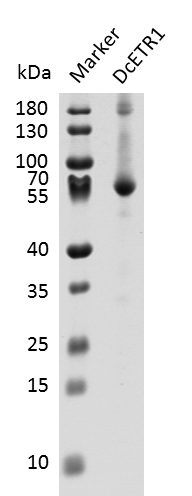
**

**Supplementary Figure S1 ⏐ Heterologous expression and purification of carnation ethylene receptor DcETR1.** DcETR1 was expressed in *E. coli* C43 (DE3) upon induction with 0.5 mM IPTG overnight at 16 °C and 180 rpm. Cells were harvested and stored until purification at -20 °C. Cells were lysed using a Cell Disruption System (CONSTANT SYSTEMS Ltd) and membrane fraction was collected by centrifugation at 40.000 x g. DcETR1 was solubilized by FosCholine 16 and purified by immobilized metal ion affinity chromatography (IMAC). M: PageRuler Prestained Protein Ladder (Thermo Scientific). DcETR1: 1 µg of purified DcETR1 was loaded on a Tris-Tricine-SDS Polyacrylamide gel and electrophoretically separated for 1 h at 30 mA. The gel was stained using colloidal Coomassie G-250.

**Figure S2**


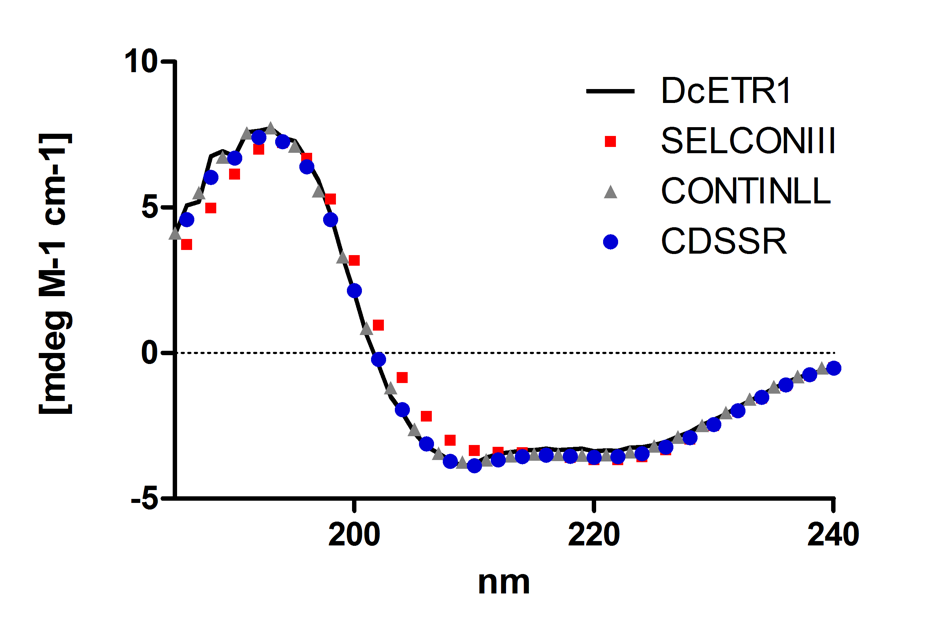


**Supplementary Figure S2 ⏐** **Circular dichroism (CD) spectrum of purified DcETR1**. Far UV-CD scan of 0.2 mg/ml purified DcETR1 in 10 mM potassium phosphate pH 7 (black line) recorded at room temperature with a JASCO model J-715 spectropolarimeter (Jasco Corporation, Groß-Umstadt, Germany). The software package CDPro was used to calculate the amount of secondary structure elements (see also **Table S1**).

**Figure S3**

**
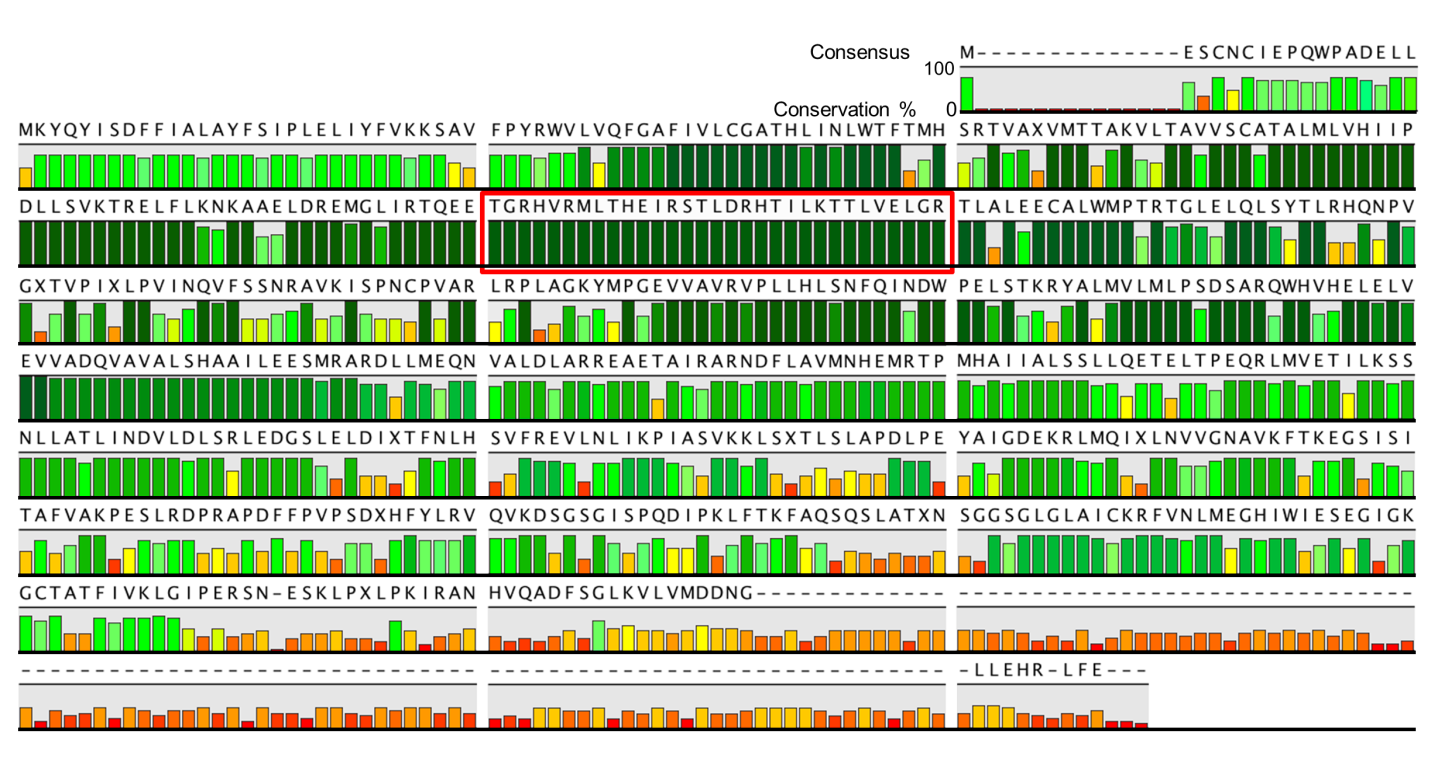
**

**Supplementary Figure S3 ⏐ Conservation analysis from complete alignment of ETR1 homologs from 17 flowering ornamentals widely used as cut flowers.** Colors show degree of conversation for each residue, dark green: 100 % conservation, dark red: 0 % conservation. The red box highlights the proposed binding site of the NOP-1 peptide in the GAF domain. For accession numbers see the Material and Methods section.

**Supplementary Table S1 ⏐ Secondary structure content calculated by SELCONIII, ContinNL and CDSSR algorithms of** **CDPro software package.** Calculated structure elements are α-Helix **H(r)**; *distorted* Helix **H(d)**, β-Strand **S(r)**, *distorted* β-Strand **S(d)**, ***Turn***, and *unordered* **(Unrd).**

| **Structure element** | **H(r)** | **H(d)** | **S(r)** | **S(d)** | **Turn** | **Unrd** |
| --- | --- | --- | --- | --- | --- | --- |
| **SELCON3** | 24.7% | 15.1% | 8.7% | 6.7% | 19.3% | 25.6% |
| **CONTINLL** | 26.4% | 16.0% | 6.7% | 5.8% | 19.4% | 25.7% |
| **CDSSTR** | 26.9% | 16.7% | 8.2% | 6.7% | 18.5% | 22.3% |
